# Early and sustained community engagement to reach unreached populations for malaria elimination in Lao People’s Democratic Republic

**DOI:** 10.1186/s40249-025-01388-4

**Published:** 2025-11-27

**Authors:** Sanjeev Ranjan Roy, Virasack Banouvong, Elizabeth Hoban, Boualam Khamlome, Keobouphaphone Chindavongsa, Inpanh Inthirath, Silivon Inthivong, Khamfeuang Sibounheuang, Khonephanom Akavong, Tran Thi Giang Huong, Rajendra Prasad Hubraj Yadav, Pascal Ringwald, James F. Kelley, Phonepadith Xangsayarath, Matthew Scott Shortus, Rita Reyburn

**Affiliations:** 1 Integrated Communicable Disease Unit, World Health Organization, Lao PDR Country Office, Vientiane, Lao PDR; 2 Center for Malariology, Parasitology, and Entomology, Ministry of Health, The Government of Lao PDR, Vientiane, Lao PDR; 3 Integrated Communicable Disease Unit, Division of Programs for Disease Control, World Health Organization, Western Pacific Regional Office, Manila, The Philippines; 4 Provincial Health Department, Ministry of Health, The Government of Lao PDR, Samakkhixay, Attapue Lao PDR; 5 District Health Department, Ministry of Health, The Government of Lao PDR, Vongsamphan, Phouvong, Attapue Lao PDR; 6 Department of Communicable Disease Control, Ministry of Health, The Government of Lao PDR, Vientiane, Lao PDR; 7 Division of Programs for Disease Control, World Health Organization, Western Pacific Regional Office, Manila, The Philippines; 8 Mekong Malaria Elimination Programme, World Health Organization, Phnom Penh, Cambodia

**Keywords:** Community engagement, Innovative approaches, Accelerator strategies, Reaching the Unreached, Malaria elimination, Lao PDR

## Abstract

Malaria incidence in the Lao People’s Democratic Republic has declined over the past 10 years. There is a continued risk of outbreaks, particularly in the Southern region, due to high-risk behaviors, primarily in remote ethnic communities among forest goers (individuals who regularly work or sleep in the forest), farmers on forest fringes, and vulnerable populations in these highly receptive areas. Conventional malaria control interventions alone in these areas are insufficient to push elimination beyond “the last mile”. In 2022, an innovative approach to accelerate malaria elimination, termed locally as “Accelerator Strategies” was implemented. Activities included targeted drug administration and intermittent preventive treatment for forest goers and mobile populations, specifically farmers on forest fringes, as chemoprevention among individuals at increased risk of malaria irrespective of infectious status. Community engagement approaches were essential to ensure participation and acceptance as the intervention requires individuals without symptoms to take medicine. Three key enablers for community participation were identified as: (1) Service delivery and community engagement by the community members themselves; (2) Strong advocacy and political commitment from senior local political leaders, and village authorities and influencers and (3) Delivering people-centered services beyond the village with granular local data on risk behaviors, population movement and geographic information system mapping. Early and sustained community engagement resulted in high coverage of the interventions and greater acceptance by the community that resulted in a decreased malaria burden.

## Background

The WHO Global Technical Strategy for Malaria calls for a reduction in global malaria incidence and mortality rates of at least 40% by 2020, 75% by 2025, and 90% by 2030 compared to the 2015 baseline; and at least 35 countries achieve malaria elimination by 2030 [24]. The Greater Mekong Sub-region (GMS) countries are committed to eliminating malaria by 2030, particularly to reduce the threat of multidrug resistance in the area [19]. In alignment with these goals, Lao People’s Democratic Republic (Lao PDR) aims to eliminate all species of malaria from the northern provinces by 2025 and nationally by 2030 [26]. Malaria cases in Lao PDR have declined over the last decade, from over 50,000 cases in 2014 to 342 cases in 2024, primarily due to the rollout and strengthening of core anti-malaria interventions (early diagnosis using rapid diagnostic tests, prompt treatment, long-lasting insecticidal nets (LLINs), village malaria workers (VMWs), and outbreak response with active case detection guided by granular and timely surveillance data) [17]. Malaria remains endemic in the southern part of Lao PDR due to high-risk behaviors, particularly among marginalized ethnic minority populations from the poorest quantile in the country [26], and Phouvong District in Attapue Province remains a significant malaria hotspot.

From 2021 to 2024, The Government of Lao PDR national malaria control program, The Center for Malariology, Parasitology, and Entomology (CMPE), with support from the World Health Organization (WHO) Lao PDR Country Office and the WHO Mekong Malaria Elimination (MME) Programme, designed and implemented the Accelerator Strategies (AS) to fast-track malaria elimination by reducing the malaria parasite reservoir and interrupting transmission in the highest-burden areas. These strategies were adapted from WHO frameworks for malaria elimination [25]. Lao PDR and Cambodia are the first countries to apply these interventions for malaria elimination in the GMS. The AS includes two forms of chemoprevention, that provide a full therapeutic course of an antimalarial medicine to high-risk populations regardless of their infection status: the first is targeted drug administration (TDA), offered to individuals in the highest-risk communities to clear the parasite reservoir, followed by intermittent preventative treatment (IPTf) for the highest risk individuals in those same communities, the forest and field goers, to prevent reinfection and reintroduction of the malaria parasite into the community. A separate manuscript on the design and implementation of the AS in Lao PDR is in development.

The AS is aligned with the WHO Global Technical Strategy for Malaria [24] to accelerate malaria elimination and the WHO Regional Framework for Reaching the Unreached in the Western Pacific (2022–2030) [27] that guides Member States to strengthen primary health care to reach the underserved populations through five key action domains: political commitment, multi-stakeholder engagement, data and evidence, community engagement as a platform to transform health services to reach the unreached, and special approaches, specific to the country needs. Early and sustained community engagement was a prerequisite for empowering communities and fostering ownership of the malaria AS interventions in Lao PDR [3, 14].

This paper describes the implementation and outcomes of AS in Phouvong District, Attapeu Province, and how community engagement ensured high-risk populations’ acceptance and participation in the AS, ultimately reducing the malaria burden in intervention sites.

### Intervention setting and malaria context

Attapue Province is in the southernmost part of Lao PDR. The southern area of Phouvong District is remote, forested, and mountainous while the northern area is flat and cultivated. The majority of the Phouvong population is from the ethnic minority group Brao (Mon-Khmer) (95% of the population) [9], with Lao as their second language [16]. Literacy is 81% among males and 71% among females and Phouvong district, with a population of 17,676 has a poverty rate of 37% and a high vulnerability index of 0.49 compared to the national vulnerability index of 0.38 [22] (Vulnerability index is a measure of the exposure of a population to various hazards, including multiple factors such as social, economic, environmental, and infrastructural factors).

Phouvong District is a critical residual *Plasmodium falciparum* and *P. vivax* hotspot due to highly receptive eco-types and a significant proportion of the population being forest-goers. Malaria is reported among all age groups and genders, although cases are predominantly among adult males. In 2019–2021, Attapue Province reported 33% (4781/14,270) of total cases in Lao PDR, and 47% (2266/4781) of these cases were reported from Phouvong district.

The populations affected by malaria are hard to reach because they live and work in the forest or forest fringe [7, 11, 15] for cultivation, food gathering, and collecting wild products for medicinal and small-scale commercial purposes. These forested areas have an abundance of efficient vectors (*Anopheles dirus*, *An. minimus*, and *An. maculatus*) for malaria transmission [10] making them extremely high-risk areas for malaria infection. Access to the areas is generally poor, on dirt tracks by motorbike or on foot. The time people spend in these areas ranges from a few days to a few months during the harvest season, and they live in temporary shelters, locally known as “Katos” or ‘scattered sites’. There are more than 140 scattered sites among the 14 AS villages (Table 1), utilized by an estimated 36% of the villages’ population. Many families possess more than one scattered site, and they may change location annually as cultivation yields are high in the first year and farmers rotate fields every 2–3 years for better yields using slash-and-burn techniques. In recent years, Phouvong district has witnessed a significant growth in cassava plantations due to increased international demand for the product. The majority of cases [57%, 160/280] in Phouvong between January and June 2022, had a travel history to the scattered sites.

**Table 1:** High-risk populations in accelerator strategy villages

District	Village	Total population of the village	No. of scattered sites	Estimated Population of the sites	Estimated length of stay of people at the sites	Malaria cases in sites (Jan-Jun2022)	Distance from main village
Phouvong	Phouhom	1191	17	541 (45%)	1 − 2 weeks	16	Ranges from 3 km − 30 km
	Vangyang	1337	12	276 (21%)	Mar to Nov	15	Ranges from 6 km − 25 km
	Ta-oum	593	11	189 (32%)	Mar to Nov	12	Ranges from 4 km − 22 km
	Vongvilainuea	802	12	126 (16%)	April to Nov	4	Ranges from 5 km − 12 km
	Vongvilai-Tai	456	1	63 (14%)	Almost whole year	2	6 km
	Vonglakhone	596	4	502 (84%)	Mar to Nov	17	Ranges from 5 km − 15 km
	Makkieng	641	11	325 (51%)	Depends on season	14	Ranges from 6 km − 24 km
	Kang	822	7	584 (71%)	Depends on season	5	Ranges from 8 km − 30 km
	Palai	759	9	317 (42%)	Mar to Nov	13	Ranges from 5 km − 18 km
	Lamong	497	6	407 (82%)	Depends on season	1	Ranges from 4 km − 12 km
	Viengxay	1737	26	706 (41%)	1 week to few months	24	Ranges from 5 km − 25 km
	Vongsamphan	2136	9	133 (6%)	Mar to Nov	14	Ranges from 2 km − 10 km
	Chiengieng	321	10	196 (61%)	Mar to Nov	13	Ranges from 5 km − 37 km
	Vongxay	582	6	113 (19%)	Mar to Nov	10	Ranges from 3 km − 6 km
Total	14	12,470	141	4478 (36%)		160	

### Accelerator strategies

The AS interventions aimed to reduce *P. falciparum* parasite reservoir in the highest-burden areas, eliminate residual *P. falciparum* hotspots, and complement, maintain, and strengthen the core anti-malaria interventions. The AS activities include community engagement and social mobilization, undertaking a census, distribution of LLINs and long-lasting insecticidal hammock nets (LLIHN) in AS villages, TDA, IPTf, and active fever screening (AFS) (Table 2).

**Table 2:** Accelerator strategy activities and corresponding political advocacy, community engagement, outcome indicators and impact on malaria cases by year

Period	Activity	2022			2023				2024	
		Political advocacy	Community engagement	Political advocacy		Community engagement		Political advocacy		Community engagement
Jan-Mar	Planning at national and subnational level	Central > subnational		Central > subnational				Central > subnational		
Apr-May	District level training and advocacy	District > village		District > village				District > village		
	Village level social mobilization		MoH, VL, VMW > Community	District > village		MoH, VL, VMW > Community		District > village		MoH, VL, VMW > Community
	Census									
	TDA			District > village				District > village		
	LLIN & LLIHN top up									
Jul-Sept	IPTF		VMW > Community			VMW > Community		District > village		VMW > Community
	Outreach for IPTF	No outreach for IPTF				VMW > Community				VMW > Community
	AFS		VMW > Community			VMW > Community		No AFS		
Apr-Sept	Data reporting									
	M&E									
Outcomes		TDA coverage = 77% IPTF coverage = 48%			TDA coverage = 91% IPTF coverage = 90%				TDA coverage = 90% IPTF coverage = 96%	
Number of cases		Pf cases = 125 Pv cases = 442			Pf cases = 26 Pv cases = 48				Pf cases = 3 Pv cases = 13	

Key: Central > subnational: advocacy from central level leaders to subnational level leaders, District > village: advocacy from district level leaders to village level leaders, MoH, VL, VWM > Community: MoH, village leader and VMW community engagement with the community, VWM > Community: VMW community engagement to community, *TDA* targeted drug administration, *IPTF* intermittent preventative treatment for forest goers, *LLIN* = long lasting insecticide treated net, *LLIHN* long lasting insecticide treated hammock net, *AFS* Active fever screening

**Fig. 1:**
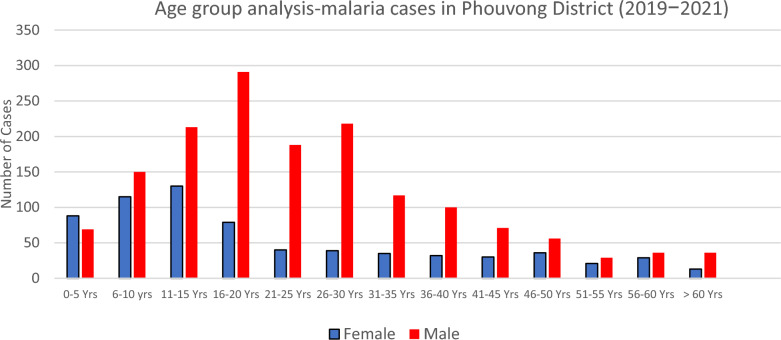
Malaria cases age-group analysis in Phouvong District, 2019−2021. Source: District Health Information System2. Yrs: Years

In 2022, 11 villages in Phouvong District were selected for the AS interventions, including peri-urban and rural villages, based on the high number of total malaria cases (prioritizing villages with *P. falciparum* cases over *P. vivax* cases) in the past three years. The target area for the AS was expanded in 2023 to include three more high-burden villages. Between 2019 and 2021, the 14 AS-targeted villages reported 85% of malaria cases in the district (1928/2266 total cases). People aged 7–60 years old were the target group for the AS interventions as they account for 88% of all malaria cases (Fig. 1).

Before each round of the TDA, community advocacy and mobilization were conducted, followed by a census of the entire population. Each year, two rounds of TDA were conducted, one month apart, for individuals aged 7–60 years old without contraindications. The TDA involved three days of the second-line treatment for *P. falciparum*: artemisinin-pyronaridine (Pyramax^®^), and a single dose of primaquine. The TDA was administered under direct observation in the villages and during outreach to scattered sites, with light snacks provided. Each TDA lasted six days in a village, during which time the team members stayed in the village. Long-lasting insecticidal nets were distributed to families, with one bed net for every 1.8 people, and LLIHNs were given to families that engaged in forest activities. The VMWs provided three monthly rounds of IPTf for forest goers following the TDA in the villages in all years of AS. In 2023, mobile malaria workers (MMWs) and kato malaria workers (KMWs) were added to the service delivery model to provide IPTf during outreach visits to the scattered sites, following low coverage of IPTf in the villages due to significant population movement to the scattered sites. Additionally, AFS was conducted every two weeks in the intervention villages and scattered sites in 2022 and 2023 but ceased in 2024 due to the low positivity rate in previous years (Table 3).

**Table 3:** Summary of Malaria Accelerator Strategies’ objectives, delivery methods and frequency of activities

	**Target population (Risk groups)**	**Objectives**	**Delivery method**	**Frequency**
Community engagement and census	Whole village population and specifically working closely with community leaders	To actively engage and ensure participation in all activities; inform planned interventions, the risks, and benefits and get feedback; strengthen good relationships, communication, and trust between the community, local leaders, and service providers. Record individual-level data and define each person’s risk group	Delivered at the village by HC staff and VMW with oversight from DAMN, PAMN, and CMPE	Formal community engagement was conducted every 6 months and at the start of each TDA Informal community engagement was conducted by the VMW every 2−4 week, during the door-to-door active fever screening. The census was conducted once
LLIN & LLIHN distribution	Whole village and field-going population received LLIN. Forest goers received LLIHN	To ensure the community has good coverage of LLIN/LLIHN	Distribution was done in the targeted villages and via outreach to scattered sites	Once
TDA	Whole village population, 7 − 60 years of age, excluding contraindications	To ensure the whole population is protected from malaria infections, eliminate *P. falciparum* and *P. vivax* in the target population. With ACD among people aged < 7 & > 60yrs to detect and treat any cases of malaria	Delivered at the village by HC staff and VMW with oversight from DAMN, PAMN, and CMPE. Via outreach to scattered sites	Two rounds, each one month apart
IPTf	People 7 − 60 years of age, excluding contraindications, which fit into one of the three high-risk groups: Forest goers Field goers; or Socialize outside at night	To protect those most at risk from infection while they are exposed and prevent the reintroduction of parasites into the village	For forest and field goers—VMWs will deliver before groups go to the forest or field. Via outreach to scattered sites	VMW should be available every day to give IPTf before people go to the field or forest In addition, for people who do not come to the VMW to receive IPTf, the VMW may give IPTf during door-to-door visits. MMW/KMW will provide IPTf monthly via outreach to scattered sites
Active fever screening	The whole village was screened for symptoms including demographic groups not targeted for TDA or IPTf	To ensure every symptomatic person is tested for malaria and treated if positive to prevent the re-establishment of village transmission	House-to-house	Every two weeks in the year 2022, changed to monthly in 2023, and stopped in 2024

*CMPE* Center for Malaria Parasitology and Epidemiology, MoH Lao, *PAMN* Provincial Antimalaria Network staff, MoH Lao, *DAMN* District Antimalaria Network staff, MoH Lao, *HC* Health Center staff, *VMW* Village Malaria Worker, *MMW* Mobile malaria worker, *KMW* Kato Malaria Worker, *SSA* Special Service Agreement staff, WHO Lao, *TDA* Targeted Drug Administration, *IPTf*: Intermittent Preventative Treatment for forest/field goers, *LLIN* Long lasting insecticide treated net, *LLIHN* Long lasting insecticide treated hammock net, *ACD* active case detection

All AS intervention data were recorded and reported to the District Health Information System, version 2 (DHIS2) by health staff in facilities responsible for the villages in their catchment area. Monitoring and evaluation were conducted by the district and province health authorities to ensure data accuracy in DHIS2 and to identify operational gaps or challenges to guide operational improvements. Pharmacovigilance was undertaken to quickly identify potential adverse events in the community and appropriate actions were taken to address the medical needs of those affected, alongside community education to mitigate any risk to the program. Mild adverse events were reported among 0.6% of participants. The most commonly reported were headaches and vomiting, with very few episodes of rash, and the teams were equipped with medicines to manage the side effects. The Knowledge, Attitude, and Practices (KAP) survey was conducted after 2022, the first year of AS implementation and the findings were used to refine the AS strategy.

For AS to be successful, community acceptance and participation were essential. Both bottom-up and top-down approaches were applied to mobilize communities and ensure their active participation. The bottom-up approach, which refers to initiatives or activities delivered in the community, was led by team members close to the community, who were involved in implementing the AS, including district and health center (HC) staff, VMWs, and local leaders. The top-down approach, which refers to initiatives facilitated by multisectoral government authorities, involved district-level leaders, including the District Governor (one of the most influential persons in the district), attending the district advocacy meetings and participating in the TDA activities in some high-priority villages. The use of the bottom up and top-down community engagement strategies are presented in Table 2. Additional political advocacy was included in 2023 and 2024 which effectively strengthened community participation, evidenced by the increase in coverage of TDA and IPTF and subsequent reduction in cases.

### Preparation phase: political advocacy and population mapping

A national launch and political advocacy were undertaken to provide information about the AS and to seek high-level support and political commitment for the AS. Meetings and consultations were conducted with provincial and district authorities to sensitize them and to aid the intervention’s planning.

**Fig. 2:**
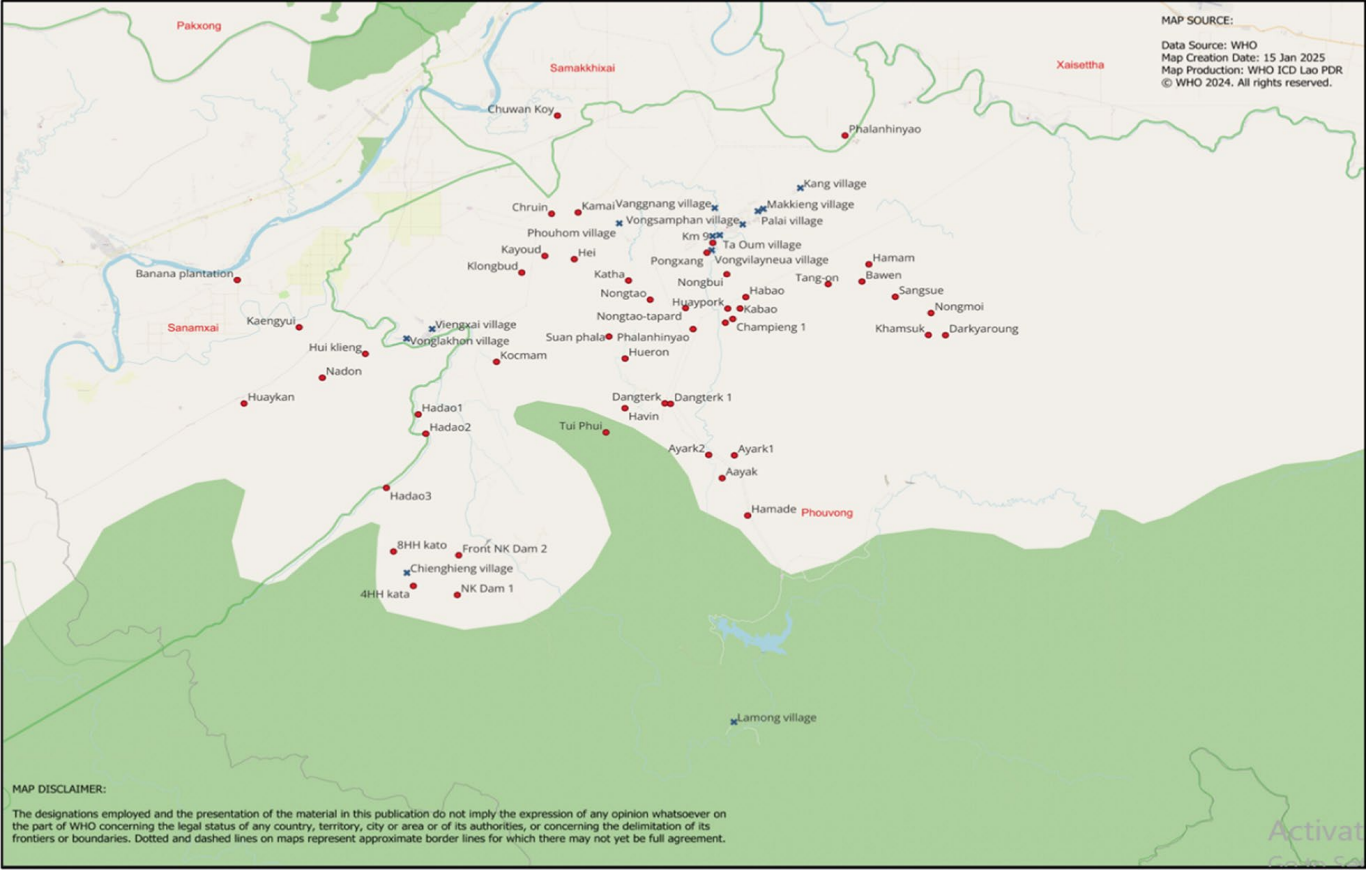
Accelerator Strategies scattered sites (red dots) and accelerator Strategies villages (blue crosses) in Phouvong District. Source World Health Organization Lao People’s Democratic Republic

In the first year (2022) of the AS implementation in Phouvong District, the teams faced major challenges in reaching the highly mobile populations, which negatively impacted the target coverage. To improve access to the unreached populations, qualitative, and quantitative data were collected on population movements and the geographic information system (GIS) locations of scattered sites’ (Fig. 2) by individuals who had strong local geographic and demographic knowledge, such as the head of villages, VMWs, and other community members. The staff from HC, district anti-malaria network (DAMN), and WHO visited villages and scattered sites and collected qualitative data through in-depth interviews from the head of villages, VMWs, and other community members on population movements to scattered sites, focusing on scattered sites with malaria cases. Data was collected on the estimated population staying in the scattered sites; the average time that villagers stay there; the distribution of cases within the sites; and the accessibility of the scattered sites to the village (Table 1).

### Community engagement during the implementation phase

To sensitize local authorities and gain their support and commitment, a one-day district-level advocacy workshop was conducted involving implementers from the district and villages, followed by two days of training for all staff. Further, on-site training on how to support work for the census and TDA was provided to VMWs, MMWs, and KMWs by technical staff at the time of activities. Community engagement via a public meeting led by village head, HC staff, district and province health staff were conducted in all target villages at the start of each TDA and during the AS implementation phase, and informal discussions were held with community members to gain their trust and support. Community engagement activities (public meeting, information education and communication through loudspeakers, posters, house-to-house interpersonal communication) were delivered in Lao and local languages by district HC staff, VMWs, and village authorities. For the success of the AS, communities needed to understand the interventions, risks, and benefits and provide feedback about the planned activities.

In target villages, most families leave their homes in the early morning for agricultural work and return in the evening. To ensure comprehensive engagement, the teams conducted house-to-house visits for AS activities in the early morning and late evening, pre-informing the villagers of planned AS activities to enhance their acceptance and participation. Teams responsible for AS implementation in the villages were further divided into sub-teams of two people, each sub-team included one technical staff and one community leader, and each sub-team covered 35–40 households. House-to-house visits provided an opportunity for interpersonal community engagement and to receive feedback from community members about the interventions.

At the end of each TDA, a review meeting was held in the district to obtain feedback from all the teams. Members of the community and implementers in each village gathered to discuss the coverage, strengths, challenges, then they developed strategies to improve the AS activities. At the end of each TDA, a summary report was submitted to the district and provincial health authorities.

### Post-implementation phase: continued political advocacy and community engagement

A KAP survey undertaken after the first year of AS implementation (2022) identified a high level of satisfaction among implementers at all levels and the community with an average satisfaction score of 8.3 − 9.7 on the scale of 1 − 10 among the implementers at all levels and an average satisfaction score of 8.8 − 9.5 among the community. The majority of respondents (82%) agreed that the AS was implemented well and achieved the target indicators. To evaluate the impact of the AS strategies and the quality of implementation, annual review meetings were conducted with the implementers including village heads and provincial authorities, allowing implementers to share achievements and challenges specific to each target village and define any adaptation and mitigation measures for the following year. The feedback loop fostered trust and ownership of the interventions at the local levels and increased support from the province and the district authorities for the interventions. It also served as a political advocacy tool to bring together national and local political leaders to review their successes and define local solutions that would strengthen activities in subsequent years.

The elements of the AS that facilitated successful community acceptance and participation are outlined in Table 4.

**Table 4:** Elements for successful community acceptance and participation in Phouvong Accelerator Strategies

Approach	Drivers of success	Impact	Considerations for sustainability	Challenges
Ownership and political commitment	Engagement of village heads and district-level authorities to champion malaria programs	Increased local accountability and ownership of health outcomes	Regular engagement with political leaders to sustain support	Changes in political focus or priorities​ in the communities
Flexibility at the local level	Teams are permitted to tailor the activities to the individual village level, accommodating cultural practices	Increased community participation and trust; improved service coverage	Maintain adaptability while adhering to national health guidelines	Balancing tailored approaches with standardized efforts; Adaptation of flexible approaches within communities
Directly observed treatment	House-to-house visits through a village-based strategy with locally trained volunteers	Improved adherence to treatment and reduced transmission rates	Scaling training programs for volunteers and providing logistical support and sufficient HR	Teams are required to work out of working hours (early morning & late evening), overcoming workforce shortages and logistical challenges in remote areas
Outreach services	Use of updated population movement data, mobilizing MMW/KMW from the same high-risk communities	Improved coverage among migrant and high-risk groups	Adequate finance and investment in outreach services	Seasonal migration, resource-intensive service delivery, and finding staff with the local knowledge and willingness to conduct outreach
Incentives	The financial and non-financial incentives for political leaders and VMWs, including transport support and recognition programmes (certificate of appreciation)	Improved motivation, increased accountability, and improved malaria services delivery through the retention of health workers	Ensure long-term financial support and continuation of community-led appreciation initiative	Potential reduction in donor funding or shifting priorities
Staff capacity	Continuous training for VMWs/MMWs on the latest malaria diagnostics and treatment protocols	Enhanced skill levels and quality of service delivery	Established long-term capacity-building frameworks and partnerships	High turnover of health staff and uneven resource distribution
System-wide approach	Prioritizing equitable access in remote areas with the future aim to integrate malaria services with health system strengthening strategies	Improved service delivery and health outcomes, reduced malaria burden in hard-to-reach areas	Continued advocacy and resource allocation for remote and community health system improvements	Geographical challenges, maintaining equity in access, coordination across sectors, and competing priorities

*VMW* Village Malaria Worker, *MMW* Mobile malaria worker, *KMW* Kato Malaria Worker

### Intervention outcomes and impact

Addressing the challenges associated with the highly mobile populations in the target villages in the first year (2022) resulted in improved coverage in subsequent years, until 2024 when AS targets were achieved (Table 5). Bottom-up community engagement efforts were crucial to foster good relationships between the AS implementers and villagers, strengthen communication and trust between the community, local leaders, and service providers, and develop ownership of the intervention by the community. Top-down community engagement efforts with the involvement of district authorities enhanced accountability and ensured that the communities had confidence in the efficacy of the interventions.

**Table 5:** Intervention outcome in Phouvong district (2022 − 2024)

Indicator		Target	Outcome					
			2022		2023		2024	
			*n*	%	*n*	%	*n*	%
Vector control	LLIN for populations sleeping in the village	** ≥ 95%**	5110	92	6550	100	3650	100
	LLIHN for forest goers	** ≥ 95%**	1833	69	2961	100	1267	100
	LLIN for field goers	** ≥ 95%**	1187	78	600	100	2209	100
TDA	TDA round 1	** ≥ 80%**	5059	73	6316	90	6454	88
	TDA round 2	** ≥ 80%**	5631	81	6702	92	6856	91
AFS	Population screened	** ≥ 85%**	21,101	48	30,060	81	*	
	Population tested	** ≥ 10%**	4753	18	4358	14		
	Test positivity rate	** < 1%**	16	0.3	2	0.02		
IPTF	Coverage	** ≥ 80%**	2393	48	10,659	90	12,547	96

*TDA* Targeted Drug Administration, *AFS* Active Fever Screening, *IPTf* Intermittent Preventative Treatment for forest/field goers, *LLIN* Long lasting insecticide treated net, *LLIHN* Long lasting insecticide treated hammock net

^*^AFS was discontinued in 2024 due to the low positivity rate of the intervention in 2022 and 2023

**Fig. 3:**
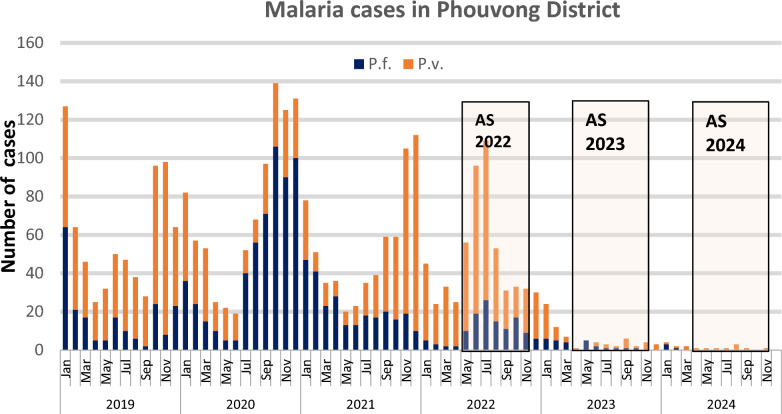
Malaria cases in Phouvong District, 2019 − 2024. Source District Health Information System2

During the period of the AS implementation, there was a 97% decrease in the total number of malaria cases, from 652 in 2021 to 16 in 2024 in Phouvong district. *P*. *falciparum* cases decreased from 265 cases in 2021 to 3 cases in 2024 (99% reduction), and *P. vivax* cases decreased from 387 cases in 2021 to 13 cases in 2024 (97% reduction) (Fig. 3). The decline has moved Phouvong District from the highest-burden district in Lao in 2021 to the cusp of elimination. A formal impact evaluation for using time series and geospatial analysis for all areas of Lao PDR conducting the AS is underway.

## Discussion

Malaria transmission in Lao PDR is highly focalized amongst ethnic minority populations who go to the forest and farms on forest fringes in remote mountainous areas to work. These populations face both physical and cultural barriers to accessing routine health services. To achieve a high coverage of the AS specialized malaria intervention, the Government of Lao PDR needed to successfully gain community trust and willingness to participate in the AS activities, as well as provide services which were easy to access. Three key enablers were identified to engage the community successfully: (1) Service delivery and community engagement by the community members themselves; (2) Strong advocacy and political commitment from senior local political leaders (district governor), and village authorities and influencers (village heads and union members) and (3) Delivering services beyond the village, which was enhanced with local granular data on risk behaviors, population movement and GIS mapping. Our work demonstrates that successful community engagement must be an iterative, adaptive, and genuine process that relies on early involvement, and strong and active participation from the community [3].

A key factor to the AS’s success is attributed to building trust, being adaptive, ensuring inclusion and representation, continuous engagement and information feedback loops with the target communities [4, 8]. This was critical as the AS interventions involved healthy individuals taking medicines. Engagement with village heads and district health authorities increased local-level accountability, ownership, and trust among the community [1]. The malaria volunteers; VMWs, MMWs, and KMWs, played a significant role in the community engagement process. The volunteers were recruited from the same community, spoke the local language, and shared the culture, traditions, geography, and population movements as the AS beneficiaries, which greatly increased access, participation, and acceptance of the AS [2, 5, 20, 21, 23].In addition, the network of well-supported malaria volunteers enabled delivering quality, equitable and accessible services to the hard-to-reach populations.

The Implementation team members stayed in the villages during the TDA. By dividing the teams into sub-teams to conduct house-to-house AS activities and targeting a small number of households, provided an opportunity to develop a good rapport with local authorities and the community, facilitating informal community engagement and subsequently achieving high coverage of AS interventions [12, 13]. Community engagement was a central feature of the AS interventions, from the planning to the post-intervention phase. The model employed throughout the AS interventions is underpinned by WHO’s operational definition of community engagement, which is “a process of developing relationships that enable stakeholders to work together to address health-related issues and promote well-being to achieve positive health impact and outcomes” [28].

Involving political leaders was vital for community participation. The district governor, as the top local official, influenced village leaders by highlighting intervention priorities and promoting advocacy. When a village had low coverage, the governor participated in activities and communicated health benefits directly, and we observed a direct increase in community engagement and coverage of interventions. The governor’s attendance at advocacy events and review meetings emphasized the significance of these interventions to village heads as well as providing an opportunity for the community leaders to provide feedback to the governor on the community experience for these activities.

Delivering people-centered services was crucial. The majority of the AS beneficiaries are involved in agricultural activities that demand hard physical labor in hot climatic conditions. Therefore, household visits were timed for outside work hours, in the early morning or evening. Directly observing treatment regimens and providing beneficiaries with light snacks before receiving medications not only reduced the side effects of the medications but, importantly, increased participants’ confidence that they had the support of health care professionals while taking medicine. The availability of health care professionals in the community is uncommon and people appreciated the quality of care they provided. To deliver outreach services to the hard-to-reach populations, granular local data on risk behaviors and population movement, with GIS mapping of locations beyond the village were required. The population and GIS mapping data collected by AS teams were used by other health services to support and plan outreach activities for other essential programs, such as childhood immunization and tuberculosis programs. This approach aligns with the WHO Framework for Reaching the Unreached [27] and the WHO Western Pacific Region’s Weaving Health for Families, Communities, and Societies in the Western Pacific Region (2025 − 2029) [29]. Both WHO guidance documents assist governments in the region to address communicable diseases and maternal, infant, and child mortality, achieve universal health coverage, and foster “grounds-up” engagement in integrated primary health care.

It is highly likely that the AS interventions, implemented to a high standard, together with strong traditional malaria elimination tools, have had a significant impact on malaria transmission in Phouvong district, as malaria case numbers had stagnated prior to the introduction of the AS in 2022. As seen in other settings, malaria chemoprevention studies demonstrate substantial impact on lowering transmission in settings when high coverage is achieved, and when chemoprevention is continued over two years [18].

Challenges faced during the AS intervention include the remoteness and inaccessibility of many areas in Phouvong district which impacted AS teams’ ability to map all the scattered sites in the AS villages, which resulted in limitations of GIS data collected in all the scattered sites. Sites with significant populations were prioritized over sites with very few people and difficult to access for operational feasibility. The use of local volunteers from within high-risk areas with good geographical and current knowledge of population movements were prioritized to achieve good coverage [6]. One limitation of this case study is the potential selection bias arising from challenges in accurately mapping mobile and hard-to-reach populations. Despite rigorous efforts to collect comprehensive data, the dynamic nature of these groups may have resulted in the underrepresentation of certain subpopulations, thereby affecting the generalizability of our findings.

## Conclusions

The success of the AS was achieved by effectively delivering services to the hard-to-reach at-risk populations, which can be attributed to engaging community members as MMWs/KMWs to design and deliver people-centered services, including outreach to hard-to-reach areas. Collection of granular and up-to-date data on population movement and location, including GIS mapping and the involvement of the district and village authorities throughout, from planning and implementation to monitoring and evaluation. These enablers enhanced community trust and increased community acceptance and participation, resulting in a decreased malaria disease burden. The findings from the AS implementation recommend early and sustained community engagement, mobilization of the community members to support the delivery of interventions, and a data-driven flexible approach to reaching the unreached.

## Data Availability

Data and materials are available upon request to the Ministry of Health, Lao PDR.

## Abbreviations

AS: Accelerator Strategies
TDA: Targeted drug administration
IPTF: Intermittent preventative treatment for forest goers
CMPE: Center for malariology, parasitology, and entomology
MME: Mekong malaria elimination programme
GMS: Greater Mekong Sub-region
VMW: Village malaria worker
MMW: Mobile malaria worker
KMW: Kato malaria worker
PAMN: Provincial antimalaria network staff
DAMN: District antimalaria network staff
HC: Health center staff
SSA: Special service agreement staff
LLIN: Long lasting insecticide treated net
LLIHN: Long lasting insecticide treated hammock net
ACD: Active case detection
AFS: Active fever screening
DHIS2: District health information system2
WHO: World Health Organization
KAP survey: Knowledge attitude and practices survey
GIS: Geographic information system
